# Pulmonary artery aneurysm associated with a bicuspid pulmonary valve: a case report of an unusual cause of atypical chest pain

**DOI:** 10.1093/ehjcr/ytag716

**Published:** 2026-09-24

**Authors:** Eryclaudia Chrystian Brasileiro Jorqueira, Vinicius de Medeiros Nobre, Nathalia Serrato Mendonça Silva, Tiago Nóbrega Morato, Antonio Aurelio de Paiva Fagundes Junior

**Affiliations:** D'Or Institute for Research and Education, SGAS 914, Asa Sul, 70390-140 Brasilia, Distrito Federal, Brazil; D'Or Institute for Research and Education, SGAS 914, Asa Sul, 70390-140 Brasilia, Distrito Federal, Brazil; D'Or Institute for Research and Education, SGAS 914, Asa Sul, 70390-140 Brasilia, Distrito Federal, Brazil; D'Or Institute for Research and Education, SGAS 914, Asa Sul, 70390-140 Brasilia, Distrito Federal, Brazil; D'Or Institute for Research and Education, SGAS 914, Asa Sul, 70390-140 Brasilia, Distrito Federal, Brazil

**Keywords:** Pulmonary artery aneurysm, Bicuspid pulmonary valve, Multimodality imaging, Case report

## Abstract

**Background:**

Pulmonary artery aneurysm (PAA) is a rare condition, most commonly associated with pulmonary arterial hypertension. Its association with a bicuspid pulmonary valve is exceptionally uncommon.

**Case summary:**

A 62-year-old woman presented with recurrent chest pain and dyspnoea, with persistently elevated D-dimer levels leading to repeated investigations for pulmonary embolism. Serial imaging demonstrated progressive pulmonary artery dilatation. Multimodality imaging identified a bicuspid pulmonary valve and a 54 mm PAA. Given aneurysm size and persistent symptoms, the patient underwent pulmonary arterioplasty with aneurysm resection. Intraoperative findings required patch enlargement with bovine pericardium. The postoperative course was uneventful, with complete symptom resolution and no recurrence on follow-up.

**Discussion:**

This case highlights a rare association between PAA and bicuspid pulmonary valve and underscores the role of multimodality imaging in diagnosis. Surgical repair may be considered in symptomatic patients and in selected patients with large or progressively enlarging aneurysms.

Learning points

- Pulmonary artery aneurysm associated with a bicuspid pulmonary valve is a rare and underrecognized condition that may present with atypical chest pain.
- Multimodal imaging plays a crucial role in diagnosis and risk stratification in cardiointensive settings.

## Introduction

Pulmonary artery aneurysm (PAA) is a rare but potentially life-threatening condition, defined as dilatation of the pulmonary artery exceeding 40 mm.^1^ It is often asymptomatic and incidentally detected on imaging; when present, haemoptysis is the most common symptom. Despite its frequently silent course, PAA carries a risk of severe complications, including rupture, which may lead to massive haemoptysis or sudden cardiac death.

PAAs occur across a wide age range and are increasingly recognized with the use of advanced imaging, particularly in patients with pulmonary arterial hypertension. They are classified as true aneurysms or pseudoaneurysms, the latter associated with a higher risk of rupture.^2,3^

The main aetiologies include pulmonary arterial hypertension, congenital heart disease, iatrogenic or traumatic injury, and infectious or inflammatory conditions.^2–4^

Prognosis depends on aetiology, aneurysm size, and complications. Surgical repair in selected patients has shown acceptable outcomes, including series reporting no early postoperative deaths.^5–7^ However, the condition remains serious due to the risk of rupture and sudden death, particularly in those with pulmonary hypertension.^1^

## Summary figure

**Figure:**
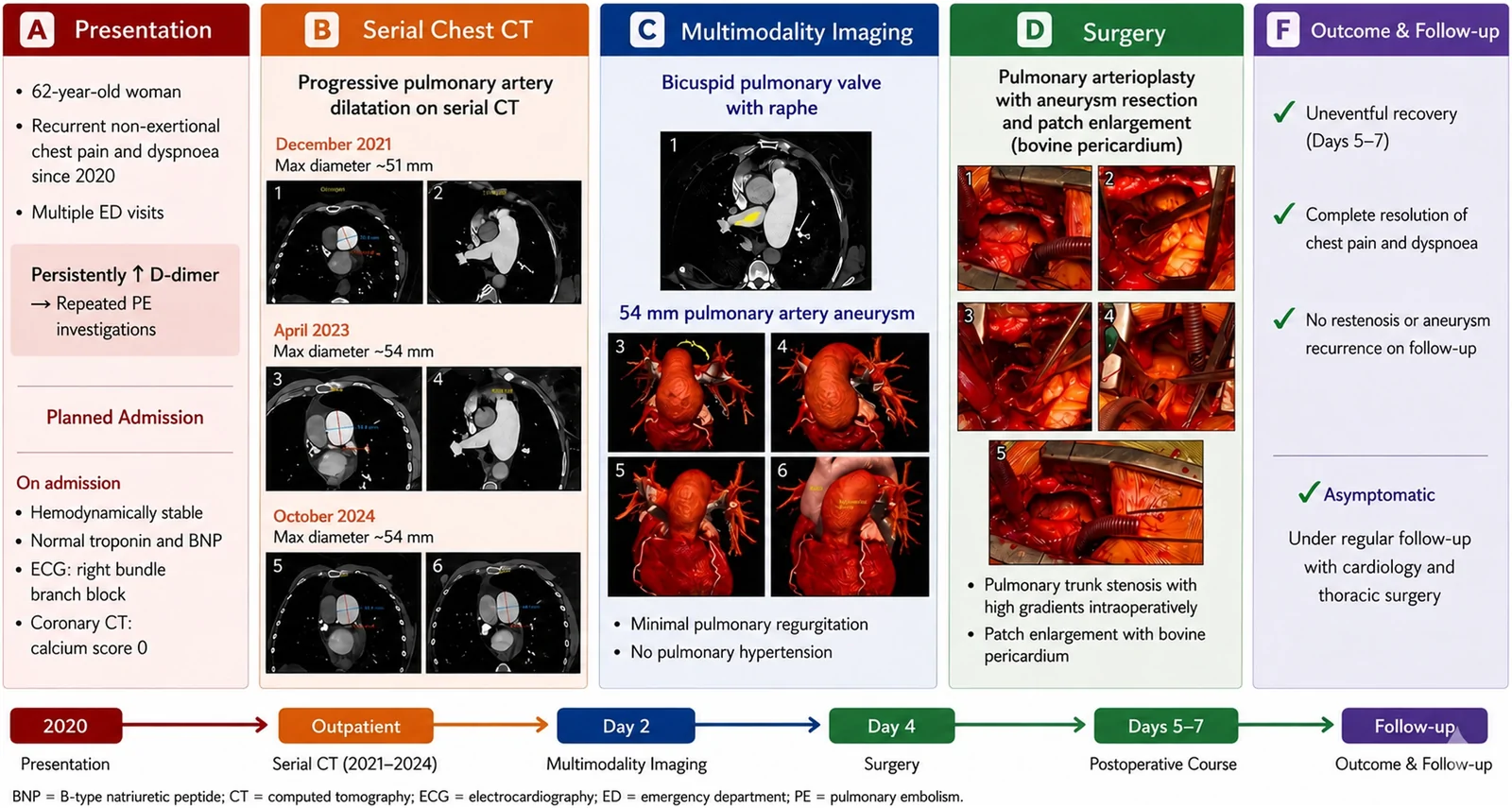


## Case presentation

A 62-year-old woman with a history of fibromyalgia, osteoarthritis, anxiety disorder, and bladder cancer under follow-up, former smoker (8 pack-years), presented with recurrent non-exertional chest pain and dyspnoea since 2020, resulting in multiple emergency department visits. D-dimer levels were persistently elevated, prompting repeated investigations for pulmonary embolism. Prior imaging since 2021 had demonstrated progressive asymmetric dilatation of the pulmonary artery; however, this finding had not been further investigated. In this context, persistently elevated D-dimer levels triggered serial diagnostic evaluations, contributing to delayed diagnostic clarification (*Figure 1*).

**Figure 1:**
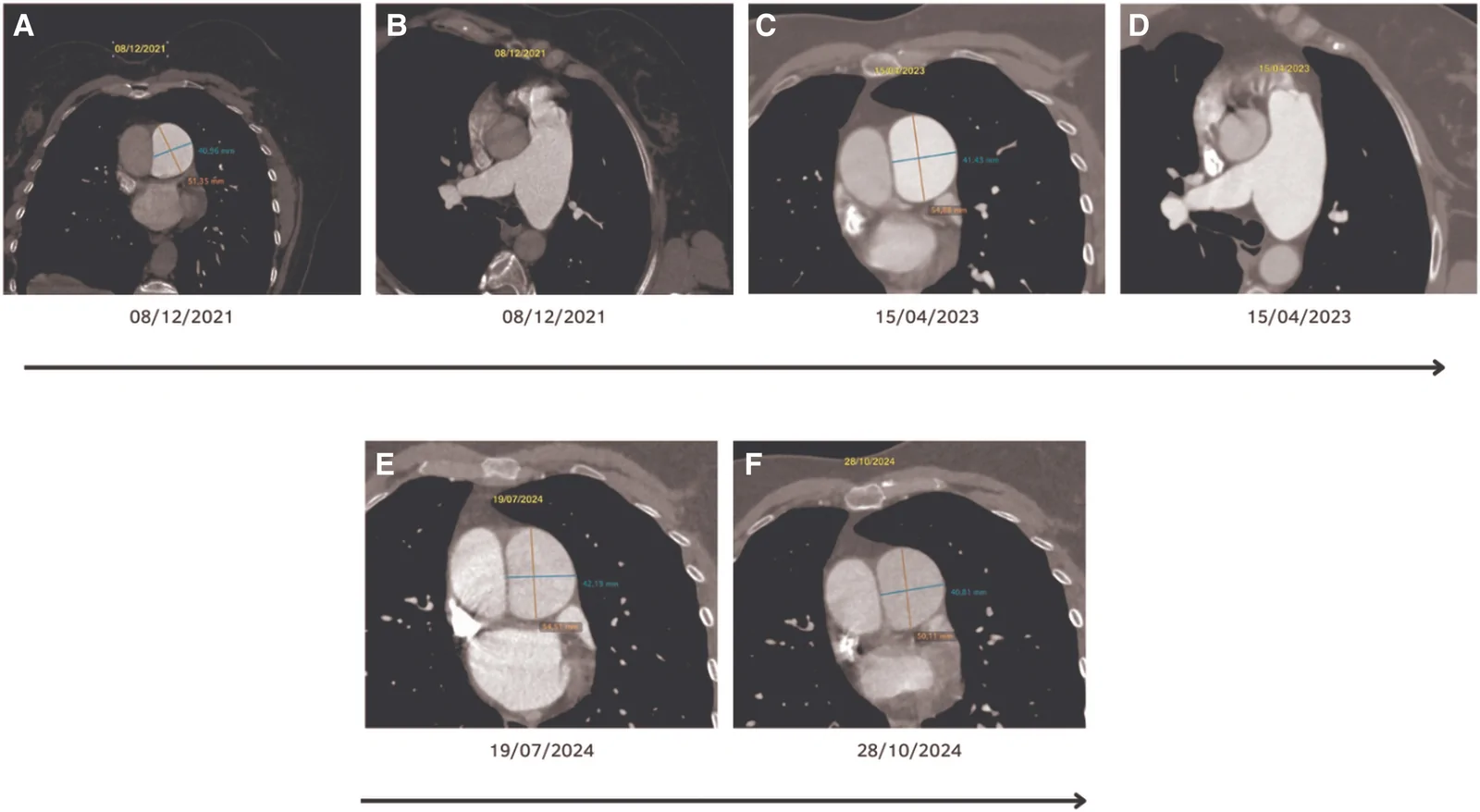
Serial contrast-enhanced computed tomography scans demonstrating progressive dilatation of the main pulmonary artery from 2021 to 2024. (*A* and *B*) December 2021: Initial CT examination showing pulmonary artery aneurysm with a maximal diameter of approximately 51 mm. (*C* and *D*) April 2023: Follow-up CT demonstrating progressive aneurysmal enlargement, reaching a maximal diameter of 54 mm. (*E*) October 2024: Subsequent CT showing persistent aneurysmal dilatation with a maximal diameter of approximately 54 mm.

Owing to poor adherence to outpatient evaluation, she was admitted for investigation of atypical chest pain. On admission, she was haemodynamically stable, with an unremarkable physical examination. Troponin and B-type natriuretic peptide levels were within normal limits, and electrocardiography showed right bundle branch block.

On Day 2, computed tomography pulmonary angiography excluded pulmonary embolism. Transthoracic echocardiography demonstrated a dilated pulmonary trunk without evidence of pulmonary hypertension, and coronary computed tomography showed a calcium score of zero. Review of prior imaging confirmed progressive asymmetric dilatation of the pulmonary artery. Multimodality imaging identified a bicuspid pulmonary valve and a 54 mm PAA with minimal regurgitation (*Figure 2*).

**Figure 2:**
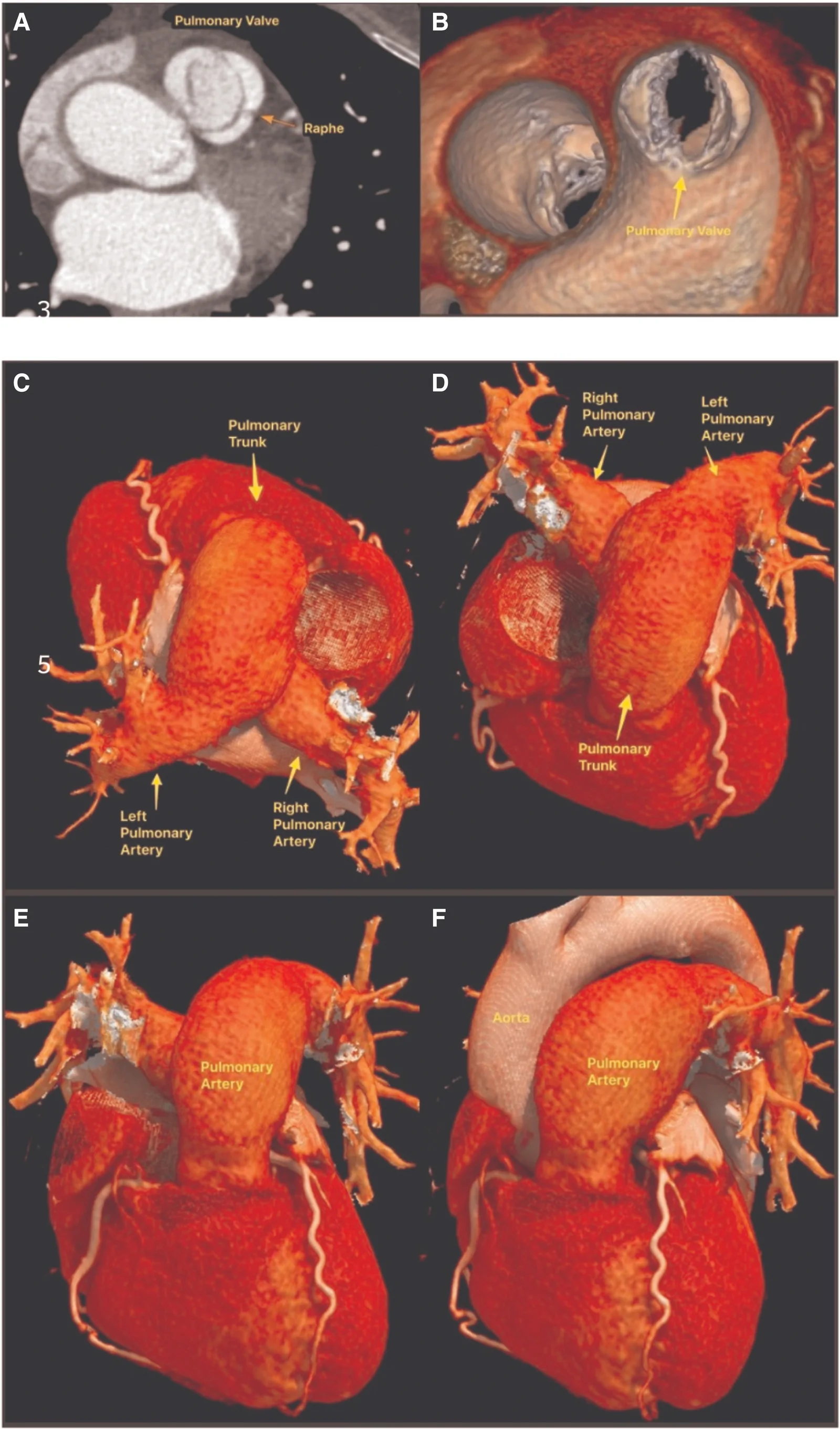
Multimodality computed tomography assessment of the bicuspid pulmonary valve and pulmonary artery aneurysm. (*A*) Axial contrast-enhanced CT image demonstrating a bicuspid pulmonary valve with a visible raphe (arrow). (*B*) Endoluminal three-dimensional reconstruction showing the abnormal bicuspid valvular orifice. (*C–E*) Three-dimensional volume-rendered reconstructions demonstrating aneurysmal dilatation of the main pulmonary artery and its branches. (*F*) Three-dimensional reconstruction illustrating the anatomical relationship between the pulmonary artery aneurysm and the adjacent ascending aorta.

A multidisciplinary team evaluated the patient. Differential diagnoses included pulmonary embolism, coronary artery disease, and gastrointestinal and rheumatological conditions, all of which were systematically excluded. Psychiatric assessment addressed anxiety-related symptoms, gastroenterological evaluation ruled out gastrointestinal causes of chest pain, and rheumatological work-up excluded vasculitis based on clinical and laboratory findings.

On Day 4, in view of aneurysm size (54 mm) and persistent symptoms, surgical intervention was recommended. The patient underwent pulmonary arterioplasty with aneurysm resection and primary suturing. Intraoperatively, transoesophageal echocardiography revealed pulmonary trunk stenosis with elevated gradients, requiring return to cardiopulmonary bypass and enlargement of the pulmonary artery using a bovine pericardial patch. Pulmonary valve repair was not required (*Figure 3*).

**Figure 3:**
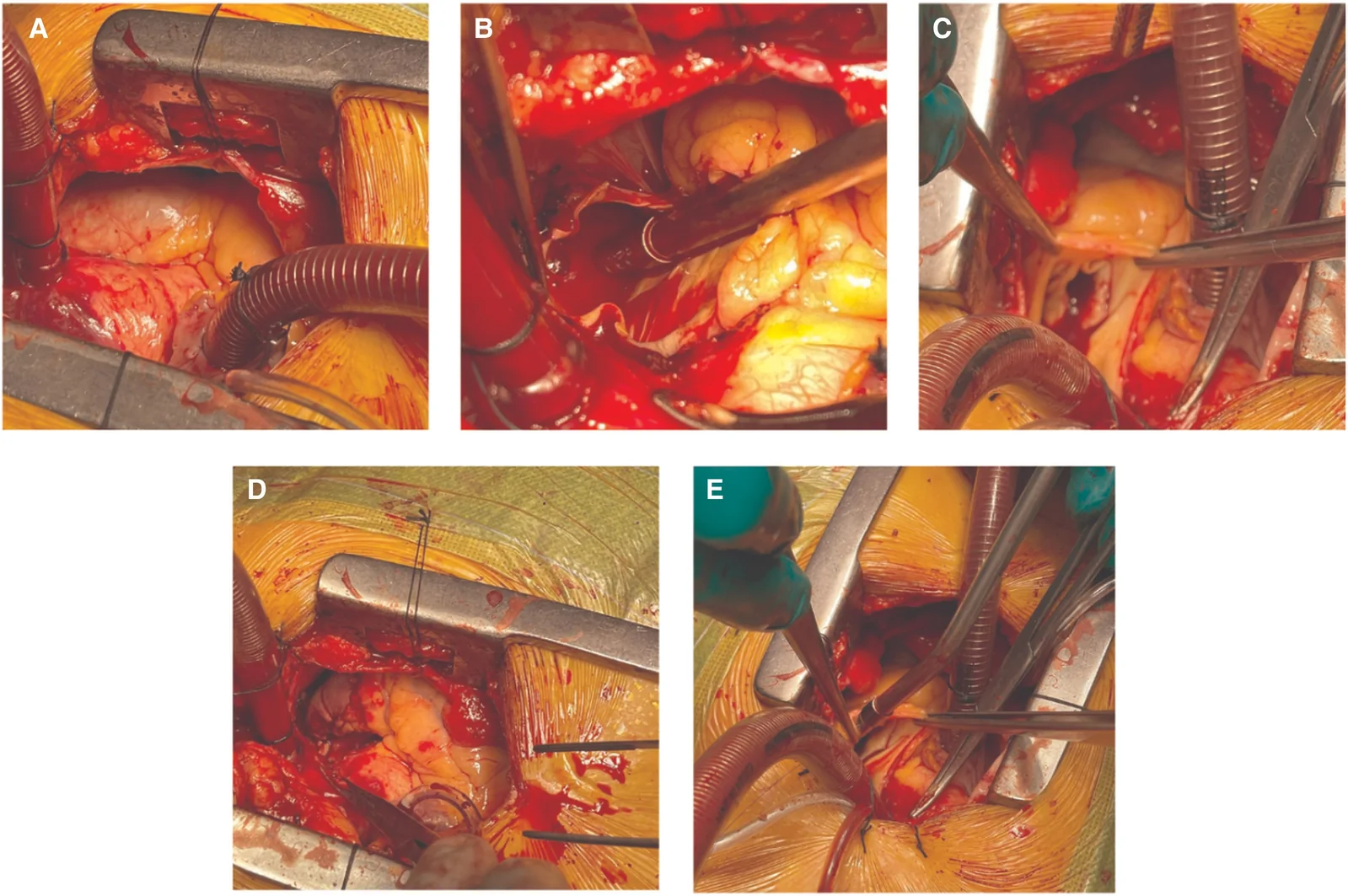
Intraoperative findings during surgical treatment of a pulmonary artery aneurysm. (*A*) Median sternotomy and pericardiotomy exposing the heart, right ventricle, main pulmonary artery, and great vessels. (*B*) Intraoperative identification of aneurysmal dilatation of the main pulmonary artery. (*C*) Dissection and mobilization of the aneurysmal pulmonary artery segment before reconstruction. (*D*) Pulmonary artery reconstruction following aneurysm resection and arterioplasty. (*E*) Final intraoperative appearance after pulmonary artery reconstruction.

Between Days 5 and 7, the postoperative course was uneventful, with complete resolution of chest pain and dyspnoea. Follow-up echocardiography demonstrated satisfactory repair, with no evidence of restenosis or aneurysm recurrence. The patient remains asymptomatic under regular follow-up with cardiology and thoracic surgery, without recurrence of chest pain, dyspnoea, or aneurysm-related complications.

## Discussion

PAA associated with a bicuspid pulmonary valve is exceptionally uncommon, highlighting the rarity and clinical relevance of this case. Clinical presentation is often non-specific, ranging from asymptomatic cases to chest pain, dyspnoea, or haemoptysis. The relationship between chest pain and PAA remains incompletely understood; however, progressive pulmonary artery dilatation may cause symptoms through local mechanical effects, and previous reports have documented symptom resolution following surgical repair.

In the present case, the coexistence of fibromyalgia and anxiety disorder introduced diagnostic complexity. Nevertheless, the progressive nature of symptoms, their temporal association with aneurysmal enlargement, and their complete resolution after surgery support a possible aneurysm-related mechanism, although definitive causality cannot be established retrospectively.

Persistently elevated D-dimer levels prompted repeated investigations for pulmonary embolism, contributing to delayed diagnosis. Although pulmonary embolism was excluded, elevated D-dimer may have reflected ongoing activation of coagulation and fibrinolysis within the aneurysmal pulmonary artery, potentially related to abnormal flow patterns, endothelial dysfunction, or mural thrombus formation. Multimodality imaging proved essential for diagnosis and characterization.^3,5^ Serial computed tomography examinations demonstrated progressive asymmetric dilatation and identified the underlying bicuspid pulmonary valve, emphasizing the importance of longitudinal imaging review.

The association between bicuspid pulmonary valve and PAA remains incompletely understood. Abnormal flow patterns and asymmetric wall stress have been proposed as potential contributors to aneurysm formation, analogous to bicuspid aortic valve-associated aortopathy, although supporting evidence remains limited. These haemodynamic alterations may explain progressive pulmonary artery dilatation even in the absence of pulmonary hypertension. The associated bicuspid pulmonary valve may also have contributed to the elevated D-dimer levels through altered flow dynamics and endothelial dysfunction.

Management remains challenging because no evidence-based guidelines define surgical thresholds for isolated PAA. Current recommendations are derived mainly from surgical case series and expert consensus.^5–7^ Although some authors have proposed a surgical threshold of 55–60 mm, a definitive size or growth-rate threshold for intervention has not been established because of the limited available evidence.^7^ Surgical intervention may also be considered in symptomatic patients or in the presence of progressive enlargement or other high-risk features.^5–7^

In the present case, persistent symptoms, progressive aneurysmal dilatation reaching 54 mm, and the potential risk of aneurysm-related complications supported surgical intervention. The patient underwent aneurysm resection and pulmonary arterioplasty; however, intraoperative transoesophageal echocardiography subsequently revealed significant pulmonary trunk stenosis with elevated gradients, requiring revision of the repair with enlargement of the pulmonary artery using a bovine pericardial patch. This unexpected finding retrospectively reinforced the appropriateness of the surgical strategy and suggested an additional haemodynamic abnormality not fully appreciated preoperatively.

Given the absence of standardized surveillance protocols for isolated PAA, serial multimodality imaging remains essential to monitor aneurysm progression and guide individualized management. This case highlights the importance of considering PAA in the differential diagnosis of unexplained chest pain, the value of longitudinal imaging follow-up, and the need for individualized treatment strategies in the absence of established guidelines.

## Lead author biography


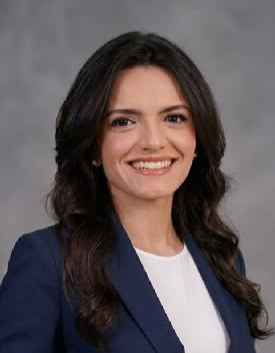
Dr. Eryclaudia Brasileiro is a resident in Critical Care Medicine at Instituto D’Or de Pesquisa e Ensino, with a strong academic focus on cardiovascular critical care. Her work integrates clinical practice and research, particularly in haemodynamic monitoring, acute coronary syndromes, and complex Intensive Care Medicine cases. She has contributed to scientific projects and case reports addressing diagnostic and therapeutic challenges in critically ill patients. Dr. Brasileiro is committed to evidence-based medicine, innovation, and multidisciplinary collaboration. Her interests include cardiointensive care, advanced monitoring technologies, and translational research aimed at improving outcomes in high-risk populations.

## Data Availability

All data underlying this article are available within the article and its supplementary material. No additional datasets were generated or analyzed for this case report.

## Notes

**Consent:** Written informed consent for publication of this case report, including clinical data and images, was obtained from the patient in accordance with committee on publication ethics (COPE) guidelines.
